# Analysis of the *Pseudoalteromonas tunicata* Genome Reveals Properties of a Surface-Associated Life Style in the Marine Environment

**DOI:** 10.1371/journal.pone.0003252

**Published:** 2008-09-24

**Authors:** Torsten Thomas, Flavia F. Evans, David Schleheck, Anne Mai-Prochnow, Catherine Burke, Anahit Penesyan, Doralyn S. Dalisay, Sacha Stelzer-Braid, Neil Saunders, Justin Johnson, Steve Ferriera, Staffan Kjelleberg, Suhelen Egan

**Affiliations:** 1 Centre of Marine Bio-Innovation and School of Biotechnology and Biomolecular Sciences, The University of New South Wales, Sydney, New South Wales, Australia; 2 J. Craig Venter Institute, Rockville, Maryland, United States of America; 3 Department of Biology, The University of Konstanz, Konstanz, Germany; 4 Institute of Infection, Immunity and Inflammation, Centre for Biomolecular Sciences, University Park, University of Nottingham, Nottingham, United Kingdom; 5 Department of Chemistry and Biochemistry, University of California San Diego, La Jolla, California, United States of America; 6 School of Molecular and Microbial Sciences, University of Queensland, Brisbane, Australia; 7 Virology Research, Department of Microbiology, South Eastern Area Laboratory Service, Prince of Wales Hospital, Randwick, New South Wales, Australia; University of Wyoming, United States of America

## Abstract

**Background:**

Colonisation of sessile eukaryotic host surfaces (e.g. invertebrates and seaweeds) by bacteria is common in the marine environment and is expected to create significant inter-species competition and other interactions. The bacterium *Pseudoalteromonas tunicata* is a successful competitor on marine surfaces owing primarily to its ability to produce a number of inhibitory molecules. As such *P. tunicata* has become a model organism for the studies into processes of surface colonisation and eukaryotic host-bacteria interactions.

**Methodology/Principal Findings:**

To gain a broader understanding into the adaptation to a surface-associated life-style, we have sequenced and analysed the genome of *P. tunicata* and compared it to the genomes of closely related strains. We found that the *P. tunicata* genome contains several genes and gene clusters that are involved in the production of inhibitory compounds against surface competitors and secondary colonisers. Features of *P. tunicata*'s oxidative stress response, iron scavenging and nutrient acquisition show that the organism is well adapted to high-density communities on surfaces. Variation of the *P. tunicata* genome is suggested by several landmarks of genetic rearrangements and mobile genetic elements (e.g. transposons, CRISPRs, phage). Surface attachment is likely to be mediated by curli, novel pili, a number of extracellular polymers and potentially other unexpected cell surface proteins. The *P. tunicata* genome also shows a utilisation pattern of extracellular polymers that would avoid a degradation of its recognised hosts, while potentially causing detrimental effects on other host types. In addition, the prevalence of recognised virulence genes suggests that *P. tunicata* has the potential for pathogenic interactions.

**Conclusions/Significance:**

The genome analysis has revealed several physiological features that would provide *P. tunciata* with competitive advantage against other members of the surface-associated community. We have also identified properties that could mediate interactions with surfaces other than its currently recognised hosts. This together with the detection of known virulence genes leads to the hypothesis that *P. tunicata* maintains a carefully regulated balance between beneficial and detrimental interactions with a range of host surfaces.

## Introduction

Bacterial surface colonisation is a process of critical ecological and economical importance in the marine environment. Initial attachment of the cells and subsequent biofilm formation are the first, important steps in a cascade of recruitment or inhibition events of secondary settling organisms, including algal spores or invertebrate larvae. On artificial surfaces like ship hulls or aquaculture infrastructure the final surface community of bacteria as well as lower and higher eukaryotes (“the fouling community”) is clearly undesirable and often causes significant cost in clean-up or replacement [1], [2]. On living surfaces like algae or invertebrates without physical or chemical defence substantial colonisation can impair host function and ultimately lead to death. However, some marine living surfaces remain unfouled in the field and the surface-associated bacterial community has been strongly implied in providing an inhibitory effect on secondary settlers. Indeed, surface-associated bacteria have proven to be a rich source of bioactive molecules that mediate the behaviour of higher eukaryotes or possess killing activities [3], [4]. Here a mutualistic relationship can be postulated where the bacterial community protects the host from fouling, while the host surface might provide nutrients and physical protection to the bacteria. However, ecological theory would also predict gradients between mutualism, commensalism and parasitism to emerge and indeed bacterial surface communities and their members can have a pathogenic effect on the host [5], [6].

Host-associated diversity is often very distinct from the planktonic diversity of the surrounding waters [4], which may be reflective of differing roles for these communities. *Pseudoalteromonas tunicata* is a model organism for microbial-host interactions on marine living surface and the production of antifouling metabolites [7]. This green-pigmented bacterium has been isolated from the surface of the marine alga *Ulva lactuca* and the tunicate *Ciona intestinalis*, and related strains from the heterotrophic, gammaproteobacterial genera *Pseudoalteromonas* are often found in association with eukaryotic hosts such as sponges, mussels, pufferfish and a range of algae [7]. *P. tunicata* produces a range of target-specific inhibitors including a large antibacterial protein [8], a small polar heat-stable anti-larval molecule [9], a putative antialgal peptide [10], a yellow-pigmented, antifungal tambjamine molecule [11], [12] and a purple pigment (violacein) that inhibits protozoan grazing [13]. This chemical arsenal has been shown to be important for the survival of *P. tunicata* in the highly competitive marine surface environment [14], [15].

To obtain a better understanding of the properties of surface-associated bacteria we present here an in-depth analysis of the genome sequence of *P. tunicata*. Comparative genomics with related strains revealed novel insight into the mode of generating genetic variations, surface attachment and biofilm formation, stress response, nutrient acquisition and ability to degrade polymers of various eukaryotic surfaces. Our analysis also suggests mechanisms for host specificity for *P. tunicata* and the potential for pathogenic interactions.

## Materials and Methods

### Genome sequencing, annotation and analysis

The genome of *P. tunicata* strain D2 was sequenced and assembled using a hybrid sequencing strategy and further details are given in Goldberg et al. [16] . Annotation was performed with the pipeline implemented by the Institute of Genome Research (TIGR), which included gene identification with GLIMMER [17], searches against PFAM [18], TIGRFAM [19], COG [20] and PROSITE [21]. All open reading frames (ORF) were manually checked, and annotations were curated using Manatee software package (http://manatee.sourceforge.net) following the curation guidelines outlined by Haas et al. [22]. Comparative analysis was performed with the Integrated Microbial Genome (IMG) system [23]. Instances of horizontal gene transfer were analysed by interpolated variable order motifs (IVOM) as described by Vernikos and Parkhill [24]. Clustered regularly interspaced short palindromic repeats (CRISPRs) were identified with CRISPR-finder [25]. The prediction of signal peptides (SP) was performed using SignalP v 3.0 [26] . The protein sequences were considered SP-positive where all the NN-scores were above the default cut-off using both softwares. The *P. tunicata* proteome was also tested for the presence of membrane spanning domains using TMHMM v 2.0 [27]. Proteins having a signal peptide were scanned for protein families against the Pfam database through global alignments (Pfam_ls) at a significance cut-off level (E-value) of 10^−5^. Phylogenetic analysis was performed by alignment of protein sequences using Clustal W [28] and neighbor-joining trees were generated with 1000 bootstraps.

### Physiological studies

To assess the ability of *P. tunicata* to grow on specific substrates, overnight cultures of *P. tunicata* were inoculated in either marine broth (Difco 2216) or marine minimal medium (3 M) supplemented with either trehalose, chitin, chitobiose or cellulose as the sole carbon source according to Stelzer et al. [29]. Cultures were incubated aerobically at room temperature (22°C) and growth was monitored by optical density (600 nm) for a period of 48 hours.

To detect phage-particles, *P. tunicata* cell-free supernatant was examined under transmission electron microscopy (TEM). The sample was placed on a carbon-coated formvar copper grid and negatively stained with 2% phosphotungstic acid for 30 sec. The grid was then examined using a Hitachi H700 TEM at an accelerating voltage of 75 kV.

Pigment production was measured spectrophotometrically using a Beckman DU 640 spectrophotometer at an absorbance of 575 nm and 425 nm for purple violacein and the yellow tambjamine pigment, respectively, as described in Egan et al. [30].

## Results and Discussion

### Overall comparison of *Pseudoalteromonas* or *Alteromononas* genomes

**Figure 1:**
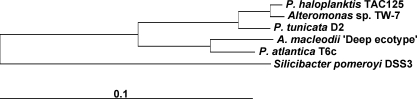
Phylogenetic tree based on 16S rRNA gene sequence of *Pseudoalteromonas* and *Alteromonas* species compared in the study. Tree was generated by maximum likelihood analysis of 1276 nucleotide positions. The sequence alignment and phylogenetic calculations were performed and manually checked with the ARB software package [97]. The 16S rRNA gene sequence of *Silicibacter pomeroyi* DSS-3 was used as an outgroup.

**Figure 2:**
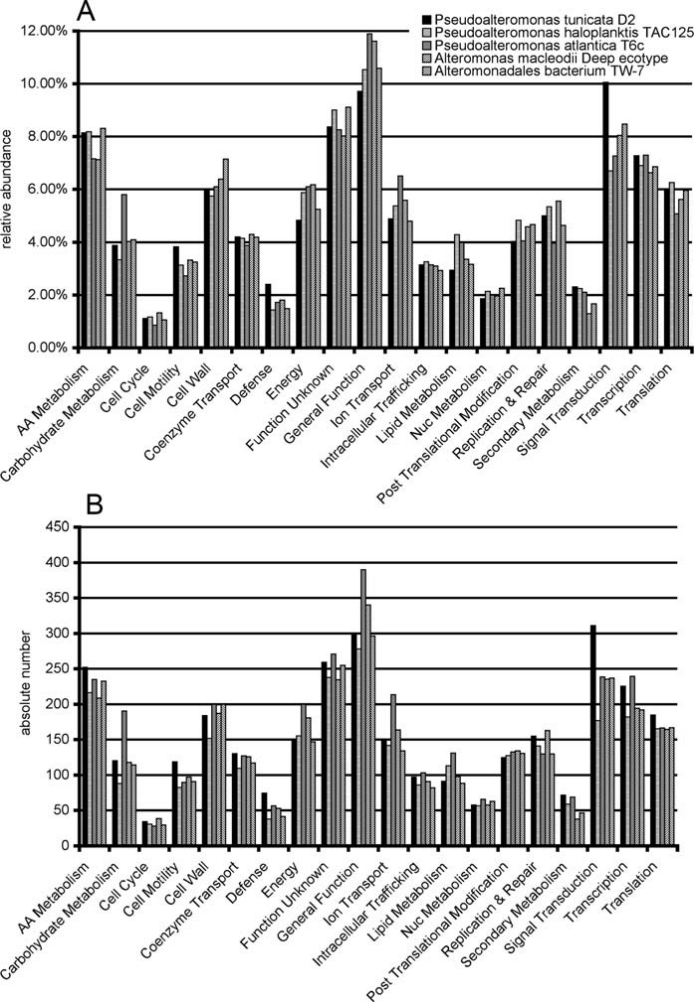
Functional comparison of *Pseudoalteromonas* and *Alteromonas* genomes. Relative abundance compared to all COGs (panel A) and absolute number of categories (panel B) for selected *Pseudoalteromonas* and *Alteromonas* species. COGs were extracted from IMG using greater than 30% identity and expectancy values of less than 10^−5^ cut-offs and assigned to functional categories.

Hierarchical clustering and principle component analysis of cluster of orthologous groups (COG) with other sequenced members of the *Alteromonadales* available in the IMG database indicate that *P. tunicata* is functionally most closely related to *P. haloplanktis*, *P. atlantica*, *Alteromonas macleodii* and *Alteromonadales* sp. TW7. General features of these genomes are given in Table 1 and their phylogenetic relationship based on the 16S rRNA gene is illustrated in Figure 1. The COG profiles between these organisms revealed however a number of COG categories that were over-represented in *P. tunicata* (Figure 2) including signal transduction mechanisms, defence mechanisms and cell motility. *P. tunicata* shows the highest proportion of genes assigned to signal transduction (10.05%) of any *Alteromondales* genome (second highest: *Shewanella amazonensis* with 8.62%; lowest: *Psychromonas* sp. CMPT3 with 5.10%). This trend is mainly caused by an over-representation of COG5000 (signal transduction histidine kinase involved in nitrogen fixation and metabolism regulation) and COG0664 (cAMP-binding proteins - catabolite gene activator and regulatory subunit of cAMP-dependent protein kinases). *P. tunicata* also has the highest value (2.4%) for the COG category defence of any *Alteromondales* genome and this is specifically due to more genes related to ABC transporter functions, including those predicted to be involved in transport of drugs and/ or antimicrobial peptides (COG0577, COG1131, COG1136, COG1566) and those related to the synthesis of potential bioactive compounds such as non-ribosomal peptide synthetase (NRPS) modules (COG1020). The abundance of genes involved in transport and expression of potential defence compounds are consistent with *P. tunicata's* successful competition on living surfaces. The major difference in the COG category cell motility is in COG0840 (methyl-accepting chemotaxis protein), where *P. tunicata* has 35 hits, which is at least twice as many as any other *Pseudoalteromonas* or *Alteromononas* species.

**Table 1:** General feature of *Pseudoalteromonas* and *Alteromonas* genomes compared in this study.

	*P. tunicata* D2	*P. haloplanktis* TAC125	*P. atlantica* T6c	*A. macleodii* Deep ecotype	*Alteromonas sp*. TW-7
Basespairs	4982425	3850272	5187005	4413342	4104952
GC percentage	40	40	45	45	40
Coding sequences (CDS)	4504	3487	4313	4163	3783
average length CDS	984	978	1051	935	972
CDS assigned to COG	3096	2639	3279	2926	2797

We also compared the genomes of *Pseudoalteromonas* and *Alteromononas* species to the predominantly planktonic dataset of the Global Ocean Survey (GOS) [31]. Using recruitment plots [31] and a 95% nucleotide identity cut-off, which is indicative for species level similarity, [32] we found 31, 59, 6, 2327 and 1269 matching reads in the GOS dataset for *P. tunicata*, *P. haloplanktis*, *P. atlantica*, *Alteromonadales* sp. TW7 and *A. macleodii*, respectively. The matches against the *Pseudoalteromonas* species are mostly in conserved regions of the chromosome (e.g. ribosomal genes) and hence reflect low gene divergence rather than true phylogenetic relatedness. The hits for *Alteromonadales* sp. TW7 and *A. macleodii* are distributed over the whole chromosome and are therefore likely due to true representation of these organisms (or close relatives) in the dataset. Over 95% of the hits in the GOS dataset come from the deeply sampled Sargasso Sea and for the same sample the abundant, free-living, phototrophic species *Prochlorococcus marinus* MED4 [33] recruits 2801 reads with the same cut-offs as above. Together this would indicate that *Alteromonadales* sp. TW7 and *A. macleodii* (or closely related organisms) constitute a significant portion of the heterotrophic population in these planktonic communities, while the three *Pseudoalteromonas* species are less prevalent.

### Mobile genetic elements

The presence of mobile genetic elements is common in bacteria, however in comparison to closely related marine bacteria they are more abundant in *P. tunicata*, where they comprise 2% of the entire genome. The *P. tunicata* genome contains one 33 kb-sized P2-like prophage, which is similar to the one found in the genome of *P. atlantica*, *Hahella chejuensis*, *Moritella* sp PE36, *P. aeruginosa* PA7, *Desulfovibrio vulgaris*, *Haemophilus influenza*, *Aeromonas salmonicida* and *Vibrio cholerae* 0395. P2-like phage is a member of the myxoviridae and is one of the 5 major prophage groups commonly detected in Gammaproteobacteria [34]. A microscopic inspection of the *P. tunicata* media supernatant in late growth phase revealed phage-like particles (Figure S1 online support material) suggesting that the *P. tunicata* P2-like phage is able to enter a lytic cycle. This observation together with the evident distribution of this prophage among marine and other bacteria could imply this phage in horizontal gene transfer that provide an adaptive advantage for the bacterial host in the marine environment or mediate acquisition of virulence genes.

There are 33 genes encoding for transposases, of which approximately half are full-length genes. Of interest is the detection of multiple copies of the insertion sequence (IS) related to the IS492 element previously characterised in *P. atlantica* [35], [36]. Control of phase variation, related to expression of extracellular polymeric substances (EPS) in *P. atlantica* has been attributed to the mobilisation of IS492. The ability of *P. atlantica* to control EPS production in this manner may have profound effects on its lifestyle, which involves the movement of cells from the seaweed surface to the water column [36], [37]. Adjacent to the IS492-related elements in *P. tunicata* are genes encoding for sensor/response regulatory proteins, a catalase enzyme and various hypothetical proteins. Other putative transposon elements (such as the IS91 and ISCps7 families) are adjacent to a relEB-like toxin/ antitoxin system, a collagenase gene and the antifungal tambjamine cluster. These transposable elements may play a role in the genetic regulation of environmentally relevant phenotypes such as environmental sensing, competition or oxidative stress resistance and their mobilisation might enable *P. tunicata* to switch between a surface-associated and a free-living form.

Integrons play a major role in genetic transfer (and spread) of antibiotic resistant gene cassettes and toxins. The genome of *P. tunicata* encodes for four putative site-specific recombinases of the IntI4 type (COG0582), which are involved in the recombination and integration of class 4 integron sequences [38]. In contrast, *Alteromonadales* sp. TW7 contains two copies of this integrase, while *P. haloplanktis*, *P. atlantica*, and *A. macleodii* possess only one copy each. Interestingly, one of these putative integrase genes in *P. tunicata* is located down-stream of the tambjamine biosynthesis cluster, which has so far not been identified in closely related strains. In addition the 30 kb sequence flanked by the gene cluster and the integrase show three regions in the IVOM analysis with signature for foreign DNA, which suggests the possibility that the antifungal characteristic of *P. tunicata* was originally derived via horizontal gene transfer. In fact the most similar cluster was identified in *Streptomyces coelicolor* A3(2), where it is involved in the production of a structural relative of the tambjamine, the compound undecylprodigiosin [39].

A clustered regularly interspaced short palindromic repeat (CRISPR) region of 5428 bp length consisting of 91 times 28 bp repeat regions with the consensus sequence (GTTCACTGCCGCACAGGCAGCTCAGAAA) and 32 bp spacer was also identified in the *P. tunicata* genome. CRISPR elements are found in approximately half of the currently sequenced bacterial and archaeal genomes and are flanked by conserved CRISPR-associated (CAS) proteins [40]. Based on the homology of the CAS proteins to DNA replicating and processing enzymes and the likely origin of the spacer regions from mobile elements it has been speculated that CRISPRs provide ‘immunity’ to foreign DNA [41]. This hypothesis was recently supported by studies in *Streptococcus thermophilus* demonstrating that the removal or addition of spacers modifies the phage-resistance phenotype of the cell [41]. Upstream of the CRISPR element of *P. tunicata* is a group of five CAS proteins with three of them having homology to known CAS classes (CAS1, 3 and 4). Perfect matches to the *P. tunicata* repeat consensus were also found in the CRISPRs of *Yersinia pestis* (strains Pestoides F Nepal516, KIM, CO92, biovar Orientalis str. IP275 and Antiqua) *Shewanella putrefaciens* 200, *Shewanella* sp. W3-18-1, *Psychromonas ingrahamii* 37, *Yersinia pseudotuberculosis* IP 31758, *Vibrio cholerae* (strains V52 and RC385), *Zymomonas mobilis* subsp. mobilis ZM4, *Photobacterium profundum* SS9, *Legionella pneumophila* str. Lens and *Erwinia carotovora* subsp. *atroseptica* SCRI1043. In contrast, CRISPR elements could not be detected in the other two *Pseudoalteromonas* genomes and in *Alteromonadales* strain TW-7. One CRISPR was detected in *A. macleodii* (55 repeats), but its repeat sequence (GTGTTCCCCGTGCCCACGGGGATGAACCA) showed no similarity to the one from *P. tunicata*.

In summary, *P. tunicata* appears to have acquired the ability to protect itself from phage infection (e.g. via CRISPRs), yet shows other evidence and mechanisms of horizontal gene transfer (e.g. integrases) and genomic variation. This would suggest the requirement for a strict preference for and regulation of the movement of mobile genetic elements for the generation of phenotypic variation and niche adaptation. The abundance of signal transduction proteins and transcriptional regulator-like proteins (COG5000; see above), many of which have currently no known function, might play an additional role in this process.

### Surface attachment and biofilm formation

Successful establishment on a host surface requires the bacterial cell to first adhere to host tissue, often followed by colonisation in the form of a biofilm. These processes have been well studied across several bacterial pathogens, however the molecular interactions occurring between marine bacteria and their hosts are not as well defined.

The genome of *P. tunicata* encodes for several cell-surface structures and extracellular polymer components known in other organisms to be important for attachment. These include genes encoding for curli, Type IV pili, MSHA- pili and capsular polysaccharide (O-antigen). Curli are proteinaceous fibres belonging to the amyloid class and can make up a major component of the extracellular matrix of bacterial cells [42]. Although curli homologs can be found in several bacterial classes, the majority of studies to date have focused on their role in cell adhesion, biofilm formation, invasion and host inflammatory response in the *Enterobacteriaceae* (namely *E. coli* and *Salmonella*) [42]. *P. tunicata* possesses all the genes required for curli production and assembly, however the gene organization differs from that described for the *Enterobacteriaceae*. Rather than the two divergently transcribed operons consisting of the major structural subunits (CsgAB) and the accessory proteins (CsgDEFG) required for transcription and assembly, the *csgABEFG* genes in *P. tunicata* appear as one continuous operon with the regulator protein CsgD located downstream and in the opposite orientation. This gene arrangement is also found in *Alteromonas macleodii*, which may indicate a relatively recent gene rearrangement event. There was no evidence for curli genes in the other two *Pseudoalteromonas* species and in *Alteromonas* sp. TW7. Studies have shown that curli play a role in the interaction between *E. coli* and *Salmonella* with plant surfaces [43], [44], raising the possibility that *P. tunicata* produces curli to attach to algal host surfaces such as *Ulva lactuca*. Thus curli may be complementary to the previously described MSHA- pili mediated attachment of *P. tunicata* cells to marine host surfaces [45].

**Figure 3:**
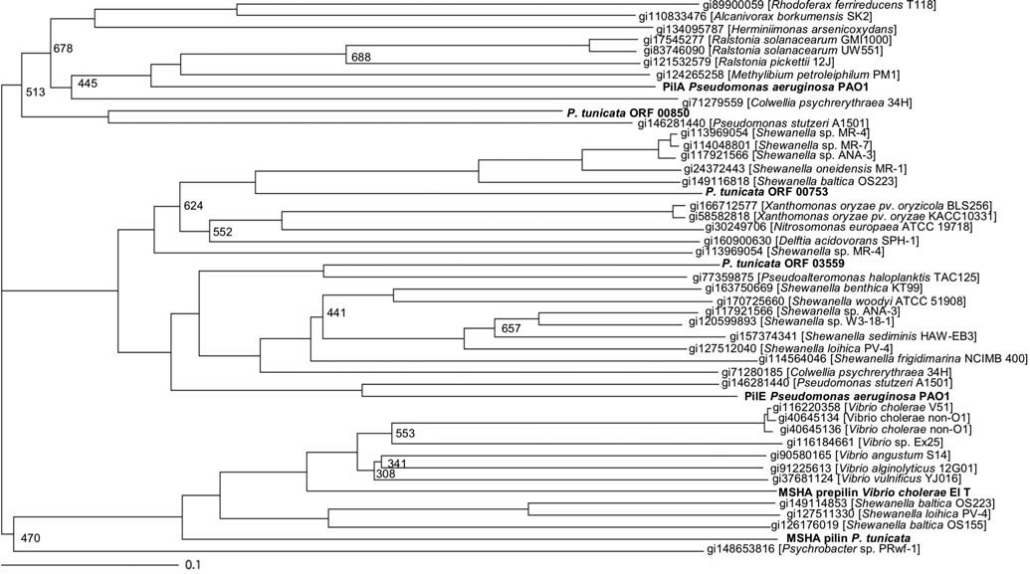
Phylogeny of pili proteins. Phylogenetic tree of pili proteins found in the *P. tunicata* genome. The pili proteins of *P. tunicata* are highlighted in bold and a specific name is given, if characterised. Other characterised pili proteins are shown in bold. The ten best blast-hits in NCBI's non-redundant database of the putative pili protein of *P. tunicata* and characterised pili proteins were used to construct the tree. Accession number and taxonomic source are shown. Bootstraps values over 750 are not shown.

The *P. tunicata* genome has nine separate gene clusters related to pili biogenesis (including the MSHA-pili). Five of the clusters contain a putative pre-pilin protein belonging to the type IVa pili characteristic of PilA or PilE proteins of *Pseudomonas* sp [46], [47]. Some of the putative structural pilin genes are quite divergent from the characterised pili and may represent new pilus-like structures (Figure 3). One of the pili gene clusters and its flanking region is highly conserved in *P. tunicata*, *P. haloplanktis* and *Alteromondales* sp. TW7 and contains homologs to the two-component response regulator system *algZ/algR*, which are involved in the regulation of alginate synthesis and pili-mediated, twitching motility in *Pseudomonas aeruginosa* [48]. Given that no alginate biosynthesis cluster is present in *P. tunicata*, we speculate that this regulatory system may only play a role in the expression of the pili cluster. Further downstream of the *algZ* is a homolog to *mviN*, a gene encoding for a membrane protein shown to be involved in the virulence in a variety of pathogens [49], [50], [51]. Upstream is a gene encoding for a homolog of ComL, a characterised lipoprotein involved in DNA uptake in *Neisseria* sp. (COG4105). Together the genes in this conserved cluster are potentially involved in colonisation and virulence and could reflect a role for *P. tunicata* as a potential pathogen.

Another interesting protein identified in the *P. tunicata* genome is LipL32, a lipoprotein so far only found in *Leptospira* species and *P. tunicata*. LipL32 is involved in adhesion to common extracellular matrix (ECM) fibres, such as collagen and laminin in *Leptospira* sp. [52]. The same study demonstrated that the *P. tunicata* homolog of LipL32 is functionally similar to that of the Leptospira protein, suggesting that this protein may have an important function in mediating interactions between *P. tunicata* and its sessile marine hosts. The fact that the tunicate *Ciona intestinalis*, a known host of *P. tunicata*, has many of the genes for the production of the ECM components mentioned above gives further support for this hypothesis [53].

The overall representation of different adhesive structures in *P. tunicata*, suggest that the bacterium can probably adhere to surfaces composed of different fibres and textures and that it possibly possesses a wider range of hosts, in addition to the previously recognised tunicates and algae.

The *P. tunicata* genome contains several enzymes for the production of a complex extracellular biofilm matrix. A capsular polysaccharide genes cluster (*cspA-D*) is present as well as a gene cluster encoding for a protein with a biosynthesis/ export domain (Pfam 02563) and similarity to the exopolysaccharide production protein ExoF from *Sinorhizobium meliloti* [54]. Downstream of the ExoF homolog lies a protein with a Wzz-like chain length determinant domain of surface polysaccharides (Pfam 02706). Upstream of the same gene is a conserved hypothetical protein that is found in the same genomic context in a range of *Vibrio* and *Alteromondales* genomes. An additional cluster of two genes with Pfam 02563 and 2706 domains is located just upstream of a Type II secretion system gene cluster and downstream of a mechano-sensitive channel protein of the MscS family. A third gene cluster contains an ExoF homolog with a Pfam 02563 domain in addition to other genes putatively involved in polysaccharide synthesis (putative ExoQ homolog, glycosyl transferase) in an arrangement unique to *P. tunicata* amongst all sequenced genomes. Together this data suggest that *P. tunicata* can produce and export a range of polysaccharides with potential role in biofilm matrix formation.

### Production of bioactive compounds and toxins

*P. tunicata* has been recognised as a bacterium rich in bioactive secondary metabolites. Analysis of the genome has revealed the genes involved in the biosynthetic pathway of previously characterised activities including the antifungal tambjamine [11], [55], and the purple pigment violacein. Genome sequencing revealed that the violacein cluster of *P. tunicata* resembled that of other violacein-producing bacteria such as *Chromobacterium violaceum*, consisting of five consecutive genes *vioA-D* and the recently described *vioE* [56]. However there is no indication for a recent horizontal gene transfer of the cluster in *P. tunicata*. Violacein has been demonstrated to have antibacterial activity and more recently to be used by biofilm-forming bacteria as a predator grazing defence strategy [13]. In *P. tunicata* it has been suggested that violacein localises to the outer membrane of the cell [13] and directly upstream from *vioA* is a gene encoding for a Multi-Antimicrobial and Toxic compound Extrusion (MATE) family efflux pump. These pumps are often used to protect the cell from damage by toxins and antimicrobial agents [57] and might provide a mechanism by which *P. tunicata* exports the otherwise toxic violacein compound.

In addition to the previously described compounds the genome analysis revealed the potential for the production of other toxins, including a putative RTX-like toxin and a toxin/antitoxin system, which is homologous to the YoeB/YefM system in *E. coli* that is believed to act as a stress regulator [58] (see below). Noteworthy is also a 61 Kb large cluster of nine predicted non-ribosomal peptide synthetase (NRPS) genes. NRPS have been identified in many microorganisms and are responsible for the production of peptides with broad structural and biological activities [59]. NRPS are modular and are often composed of an adenylation (A) domain for substrate recognition, a peptidyl carrier protein (PCP) domain that holds the activated substrate, a condensation (C) domain for peptide bond formation and a thioesterase (T) domain for termination of peptide synthesis [59]. All nine NRPS within this cluster in the *P. tunicata* genome have putative AT, C and PCP domains with one having a T domain. A second T domain containing protein is located down stream (in opposite orientation to the nine NRPS) and lies in a putative operon with a two-component regulatory system, which might be involved in the expression of this terminase in response to environmental factors. The exact nature and regulation of the compound produced by this NRPS is currently under investigation.

### Stress response

A common feature of all bacteria is their ability to sense and respond to adverse environmental conditions. Besides having all of the features for carbon and nutrient starvation as described for typical copiotrophic organism (RpoS, RelA, universal stress protein E, starvation stringent proteins and phage shock proteins) the genome of *P. tunicata* also encodes for a large number of proteins involved in oxidative stress and iron homeostasis. *P. tunicata* has four antioxidant proteins of the AhpC/Tsa family, including catalase and superoxide dismutase, in addition to an organic hydroperoxide detoxification protein and an alkyl hydroperoxide reductase, which protect against killing and DNA peroxide derived damage, respectively. Key regulators of the oxidative stress response are also present (such as SoxR). In contrast to these protection mechanisms there is an apparent absence of functions that result in the production of reactive oxygen species (ROS), except for the antibacterial protein AlpP (see below). *P. tunicata* is lacking the otherwise ubiquitous molybdopterin metabolism and several of the genes encoding for proteins that utilise this co-factor for the generation of ROS species (eg. xanthine oxidase). This feature has also been identified in the psychrophilic bacterium *P. haloplanktis* as an important strategy for reducing the effect of oxidative stress, which is increased at low temperature [60]. A high number of proteins involved in oxidative stress protection may be typical for bacteria associated with a eukaryotic host, as a common defence strategy used by a range of plants and animals is the production of oxidative bursts [61]. The oxidative stress proteins may also play an important role in protecting *P. tunicata* against its own antibacterial protein AlpP. AlpP functions as a lysine-oxidase resulting in the production of hydrogen peroxide that kills other bacteria, but also *P. tunicata* cells themselves in the centre of biofilm microcolonies [62]. A fine-tuned and differential response to hydrogen peroxide is clearly required to prevent or facilitate cell death of kin in these situations.

We also identified several genes involved in heavy metal detoxification, including *cutA* and *czcA/czcB*, which encode for a heavy metal efflux pump and are important for resistance to cobalt, zinc and cadmium [63], [64]. Heavy metal resistance has been described to occur in some marine costal isolates [65] as well as in marine *Alteromondales* species [66], [67] and might provide protection against sporadic or permanent influx of contaminations from land run-off.

### Polymer metabolism and its implication for host-bacteria interactions

**Figure 4:**
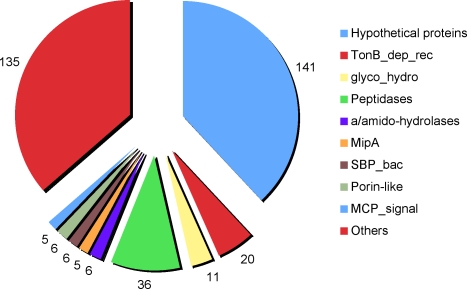
Functional properties of predicted *P. tunicata* secretome. Major protein families identified in the secretome of *P. tunicata* according to the Pfam database. Hypothetical proteins (141 entries) did not match to any HMM in the Pfam database; 20 proteins have the TonB-dependent receptor domain (TonB_dep_Rec); the glycolytic hydrolases (‘glyco_hydro’) group contains 11 proteins in total; 36 proteins have HMM domains that are found in different peptidases; ‘a/amido-hydrolases’ refers to alpha- and amido-hydrolases with 6 proteins in total; ‘MipA’ refers to Mlt-interacting protein like sequences with 5 sequences; ‘SBP_bac’ for extracellular solute binding protein includes SBP_bac_1 (1) and SBP_bac_3 (5); ‘Porin-like’ refers to the HMMs for OmpA (2), OmpH (1), OmpW (1), Porin_O_P (1) and Porin_1 (1); ‘MCP_signal’ refers to methyl-accepting chemotaxis protein (5); and ‘Others’ refers to all the other sequences that matched to different protein families in the Pfam database (135 entries).

The *P. tunicata* genome analysis indicated a niche adaptation for the acquisition of substrates for growth from the extracellular digestion of surface-associated polymeric substances and uptake and utilization of their respective monomers. Degradation of organic matter in the oceans is fundamental for the cycling of elements and normally undertaken in the pelagic zone by bacteria attached to organic aggregates [68]. Members of the Cytophaga-Flavobacterium group are amongst the most commonly found aggregate-associated organisms involved in organic matter degradation [69], [70]. According to Pfam categories the largest group of characterised proteins in the *P. tunicata* secretome are hydrolytic enzymes (Figure 4), suggesting that the bacterium is an efficient degrader of complex organic matter in the marine environment and thus may play a similar role as the CFB group on surfaces. In detail, the *P. tunicata* secretome consists of 371 predicted signal P containing proteins, and is approximately three-fold larger than that of *P. atlantica* and *P. haloplanktis* with 118 and 110 proteins, respectively. Overall the proportion of Sec-secreted proteins encoded by *P. tunicata* (8.5%) was similar to bacteria known to transport a large number of proteins to the extracellular environment. For example, *B. subtilis* is able to secrete nearly 170 proteins (∼4% of proteome) across the plasma membrane via the Sec translocation alone [71],while common plant pathogens encode about 4% to 11% of signal peptide containing proteins in their genome [72].

**Figure 5:**
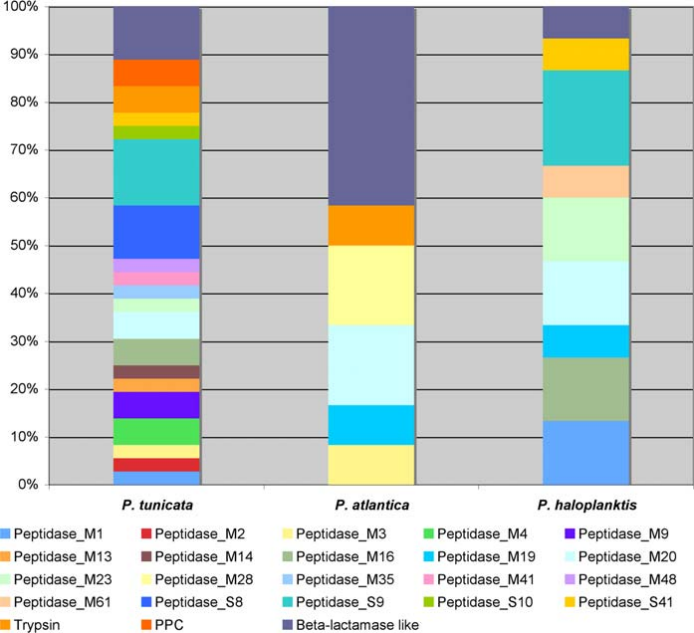
Peptidases in predicted secretome of *P. tunicata.* Comparison of the peptidase profiles in the secretome of the three sequenced *Pseudoalteromonas* species. The total number of proteins that belong to the peptidase group is 36 in *P. tunicata*, 12 in *P. atlantica* and 15 in *P. haloplanktis*. The colour bars represent the percentage of each subgroup of peptidases. Details of each peptidase family type can be found at http://merops.sanger.ac.uk.

The proteolytic potential of the *P. tunicata* secretome was high in comparison to *P. haloplanktis* and *P. atlantica*, with the identification of at least 36 peptidases (Figure 5). Two peptidases encoded in the *P. tunicata* genome match to collagenases in *Vibrio* sp. and *Clostridium* sp., and are not found in *P. atlantica* or *P. haloplanktis*. Collagen types I and II are the most abundant in cartilages of vertebrates, while marine invertebrates (including chordates) have a type of collagen similar to collagen type II [73]. Another secreted, proteolytic enzyme is a putative cyanophycinase, which degrades the amino-acid polymer cyanophycin, an important intracellular nitrogen-storage polymer predominantly found in cyanobacteria [74].

With respect to the saccharolytic potential of the *P. tunicata* secretome, extracellular digestion of the widespread carbon-storage polymer glycogen and starch was represented by sets of *alpha*-1,4 and 1,6-glucosidase and maltodextrinase genes, for which representative sets were also found in the other sequenced *Alteromonadales* genomes, but in lower abundance and degree of clustering with related gene functions, e.g. transport, intracellular metabolism, and regulation.

The *P. tunicata* genome possesses furthermore an array of genes for the digestion of chitin (poly *beta*-1,4 acetylglucosamine), an important structural element of fungal cell wall and arthropod exoskeletons frequently found in the marine environment. Growth experiments confirmed the ability to utilise chitin and chitobiose (data not shown). For binding of chitin and digestion into oligosaccharides and chitobiose, a *chiABC* gene cluster similar to the one of *Pseudoalteromonas* sp. strain S91 [75] was found, in addition to five other candidate genes for chitinase and chitin-binding function. Chitinolytic activity is common in surface-associated bacteria, such as *Vibrio* species [76], however no chitinases were found in the genomes of *Alteromonas meacleodii* and *P. haloplanktis*, *P. atlantica* and *Alteromonadales* sp. TW7. One predicted *P. tunicata* chitinase contained the unusual catalytic motif for glycoside-hydrolase family 19 chitinases, which are primarily found in plants for defence against fungal and insect pathogens [77]. This chitinase also is more similar to eukaryotic sequences (46% identical to *Zea diploperennis*) than to other prokaryotic sequences (highest identity of 39% to *S. coelicolor* A3).

For extracellular digestion of chitooligosaccharides and of chitobiose into acetylglucosamine, at least six predicted *beta*-hexosaminidase (EC 3.2.1.52; chitobiase) genes were identified, which were not present in any other sequenced *Alteromonadales* genome. Additionally, at least three extracellular polysaccharide deacetylases (COG0726) allow *P. tunicata* to release acetyl units from polysaccharides such as chitin, and two of these deacetylases had no homologs in any other *Alteromonadales* genome. Furthermore, chitin degradation might be controlled by homologs of the CdsS/CdsR two-component regulator system, which in *Pseudoalteromonas piscicida* strain O-7 was characterized to modulate the expression of chitin degradation [78], [79].

These results indicate a unique niche adaptation of *P. tunicata* for chitin degradation, and imply that chitin degradation is possibly a highly regulated and surface-associated trait. To further investigate this, we grew *P. tunicata* in liquid cultures with insoluble chitin and observed growth predominantly associated with the chitin particles. *P. tunicata* also demonstrated a reduction in the expression of the anti-protozoal compound violacein when chitin was available as sole carbon source (data not shown) hinting towards a complex regulatory network involving biopolymer degradation and inhibitor expression. In fact, *P. tunicata*'s regulated capacity of binding and degrading chitin might be regarded as a virulence trait, as chitinolytic bacteria have been associated with pathogenicity towards marine crustaceans [80], [81]. In addition, chitin degradation might accelerate the damage caused by the antifungal activity of *P. tunicata*, or a homolog to cyanophycinase and glycogen/ starch degradation might facilitate nutrient scavenging from bacterial cells lysed by AlpP. Together these findings imply, that *P. tunicata* does not only use its antimicrobial traits to out-compete other organisms for surface space, but might also effectively utilize the polymeric biomass of the carcass of competitor organisms for its own growth.

Cell wall components of plant cells, in particular the *beta*-linked polysaccharides cellulose and xylan, are also potential substrates for an organism that preferentially lives on plant surfaces, including marine algae. Based on physiological and genetic analysis *P. tunicata* is not able to degrade cellulose. In particular, the putative extracellular endo-cellulases (EC 3.2.1.4; *beta*-1,4-endoglucan hydrolase) and endo-xylanases (EC 3.2.1.8; *beta*-1,4-endoxylanase) identified in *P. haloplanktis* TAC125 and *P. atlantica* T6c were absent in the *P. tunicata* genome. However, *P. tunicata* possesses an homolog to the *Pseudomonas fluorescens* subsp. *cellulosa* exo-cellohexanase (EC 3.2.1.74, *beta*-1,4-exoglucosidase), which catalyses the degradation of oligosaccharides up to the length of cellohexose, but not cellulose and xylan. Also absent were enzymes for the hydrolysis of agaropectin, agarose, inulin, levan and pectin. Oxygenase genes predicted for the degradation of poly-aromatic compounds, such as in plant-derived lignin or humic substances, were also underrepresented, or absent, in the *P. tunicata* genome. In contrast, *P. tunicata* has complete sets of genes for central metabolic pathways that confer interconversion of the monomers derived from extracellular polymer degradation and for supporting the anabolic pathways, including the intracellular conversion of chitin-monomer N-acetylglucosamine via fructose-6-phosphate.

An obvious lack for the extracellular degradation of plant or algal associated polysaccharides is consistent with our understanding that *P. tunicata* has no observable, negative effect on algal host surfaces (such as *U. lactuca*). Also noteworthy is that the other recognised and unaffected eukaryotic host for *P. tunicata*, the tunicate *C. intestinalis*, is the only known animal that performs cellulose biosynthesis and incorporates cellulose into a protective coat [82]. Clearly, in the environment these hosts tissue can be damaged e.g. by other bacteria through polymer-degrading enzymes, and in this situation *P. tunicata* is likely to benefit from the decay of its host through some of its oligo-saccharide or mono-saccharide utilising pathways.

*P. tunicata* is well equipped to convert the acquired carbon substrates into intracellular storage polymers, through intracellular starch synthesis, a trait which seems widespread in *Alteromonadales* and *Vibrio* genomes. Glycogen and starch production is conferred through a glycogen synthase gene as part of a predicted six-gene operon including glycogen-branching enzyme. This gene organization is conserved in *Alteromonadales sp*. TW7, and in *Shewanella* and *Saccharophagus* genomes (*Alteromonadales*), but not in the other *Pseudoalteromonas* genomes Glycogen and starch synthesis is likely to be expressed in *P. tunicata* during imbalanced supply of essential nutrients, which in the marine habitat is most likely caused by phosphorous-limitations (see below). Mechanisms of polyphosphate-storage are absent in the *P. tunicata* genome supporting the notion that stored carbon is crucial to make opportunistic use of short-term available phosphorous pools. This picture fits the general opportunistic life style of the genus *Pseudoalteromonas* and might explain the comparative ease with which they can be cultured [83].

### Competition and acquisition of nutrients

Low phosphorous levels have been shown to be one of the main growth-limiting factors in the marine environment. Indeed in the open ocean phospholipids and nucleic acids appear to be the primary reservoirs of low- and high-molecular weight dissolved organic phosphorus (DOP). Additional phosphorous can be available in the form of surface-associated, particulate organic phosphorus (POP), bound for example to plant or biofilm surfaces [84], [85], [86]. The *P. tunicata* genome encodes high affinity phosphate transport systems (PstABC and PstS) and is well equipped to release phosphates from extracellular phosphoesters, as it encodes for at least five extracellular alkaline phosphatases as well as a number of extracellular nucleases and phospholipases. Interestingly, no gene for organophosphonate C-P lyases was identified (e.g. no Phn complex). This indicates that phospho(di)esters, rather than phosphonates (e.g. ciliatine), represent relevant sources of additional phosphorous in the habitat of *P. tunicata*.

Despite this array of enzymes for phosphate acquisition, phosphate starvation might be still of ecological relevance for *P. tunicata* and appear to be under complex regulation. During growth experiments we observed a link between phosphate starvation and pigment/ bioactive production in *P. tunicata* i.e. phosphate starvation during logarithmic growth of *P. tunicata* results in the early expression of pigments and bioactive compounds. Under the same conditions a non-pigmented mutant of the ToxR-like regulator WmpR demonstrated that pigment production could be induced upon phosphate, but not carbon or nitrogen starvation [87]. These results indicate the presence of a secondary regulator of the synthesis of bioactive compounds and pigments, which is activated by phosphate starvation. We identified in the *P. tunicata* genome homologs to the two-component regulatory system PhoR/PhoB, which has been shown in a number of bacteria to modulate gene expression in response to phosphate starvation [88]. The presence of the PhoR/PhoB proteins is likely responsible for the observed increase in pigment expression under phosphate starvation and recent reports have shown that antibiotic biosynthesis is negatively regulated by phosphate via the PhoR/PhoB system [89]. On marine surfaces, *P. tunicata* may be experiencing phosphate limitation, particularly in high-density consortia biofilms, and up-regulation of both bioactive compounds and phosphate acquisition could be an effective strategy to dominate competing organisms.

Iron is another limiting nutrient in the marine environment and the *P. tunicata* genome shows adaptation to this situation by a range of siderophore-dependent mobilisation and uptake systems. A large range of TonB-dependent siderophore receptors (TBDR) were predicted in the *P. tunicata* genome, such as homologs to the well-characterised TonB-dependent ferric vibriobactin/enterobactin siderophore receptors ViuA and VuuA of *V. cholerae* and *Vibrio vulnificus*, the TonB-dependent catecholate siderophore receptor Fiu of *E. coli*, and the TonB-dependent ferric enterochelin siderophore receptors like IrgA of *V. cholerae* and CirA of *E. coli*. Surprisingly, no genes for the synthesis of these types of siderophores were found in the genome. *P. tunicata* might only utilise these TBDR to scavenge siderophores released from other organisms. Alternatively, the TBDRs might be involved in carbohydrate scavenging as recently suggested for some phytopathogenic and aquatic bacteria [90]. However, *P. tunicata* appears to produce at least one siderophore itself as indicated by the presence of a biosynthetic gene cluster for an aerobactin-like siderophore. Aerobactin synthesis proceeds from lysine and citric acid via L-lysine 6-monooxygenase (EC 1.14.13.59), N6-hydroxylysine O-acetyltransferase (EC 2.3.1.102), and aerobactin synthase (C-N ligase, EC 6.3.2.27), and these functions were located in *P. tunicata* in a predicted four-gene operon, which is also found in *Alteromonadales* TW7, but not in other available *Alteromonadales* genome sequences. More specifically, a bifunctional enzyme in *P. tunicata* is predicted to account for both the N6-hydroxylysine O-acetyltransferase (N-terminal half) and aerobactin synthetase (C-terminal half) activities. A homolog of this bifunctional enzyme is also present in *Alteromonadales* sp. TW7 (ATW7_00745), *Photorhabdus luminescens* (CAE17002) and *Chromohalobacter salexigens* (YP_573109). The gene cluster also encodes a multidrug resistance efflux pump (COG0477), which might play a role in siderophore export.

Urea is ubiquitous in nature and many microorganisms utilise urea as nitrogen source. The *P. tunicata* genome encodes for a putative urea transporter permease of the Yut protein class, which has been characterised in *Yersinia* species, however does not possess a urease. Instead, urea is converted to ammonium and carbonate through a urea carboxylase and an allophanate hydrolase [91]. This is similar to recently described *Roseobacter* genomes that are also often found in association with host surfaces [92].

### Conclusion

The *P. tunicata* genome reflects an adaptation to successful persistence and competition on marine surfaces. The potential for *P. tunicata* to benefit from the decay of host tissue without causing direct harm, together with the production of inhibitory compounds against other colonisers, can give *P. tunicata* a selective advantage within the highly competitive surface environment. In addition, the capacity of *P. tunicata* to bind and degrade chitin-based oligosaccharides may suggest an expansion of its host range beyond that of the currently recognised algal and tunicate hosts. Interaction with a variety of hosts might also be facilitated by the range of surface structures (e.g. pili, curli) available to *P. tunicata*.

In contrast to mutualistic host associations, the presence of genes homologous to virulence traits of characterised pathogens raised the interesting speculation that *P. tunicata* could act as an opportunistic pathogen. Microbial diseases of marine organisms are increasingly being identified and there is now strong evidence that many of these disease progressions are induced by environmental factors [93]. Characteristic traits of pathogens such as the production of toxins, pili, capsule polysaccharides and siderophores have been suggested to also improve bacterial fitness toward typical environmental stress conditions [94], [95]. Therefore the study of such “dual function” traits in model non-pathogenic host associated microbes such as *P. tunicata* will play an important role in our overall understanding of the emergence of new microbial diseases.

## Supporting Information

Figure S1 Phage particle of P. tunicata. Transmission electron micrograph of phage-like structures observed in the spent medium of Pseudoalteromonas tunicata. Bar = 200 nm
(4.80 MB TIF)
