# Supplementary figures and images for: Analysis of the Pseudoalteromonas tunicata Genome Reveals Properties of a Surface-Associated Life Style in the Marine Environment
Source: PLoS One. 2008 Sep 24;3(9):e3252. doi: 10.1371/journal.pone.0003252 (PMC2536512; doi:10.1371/journal.pone.0003252)

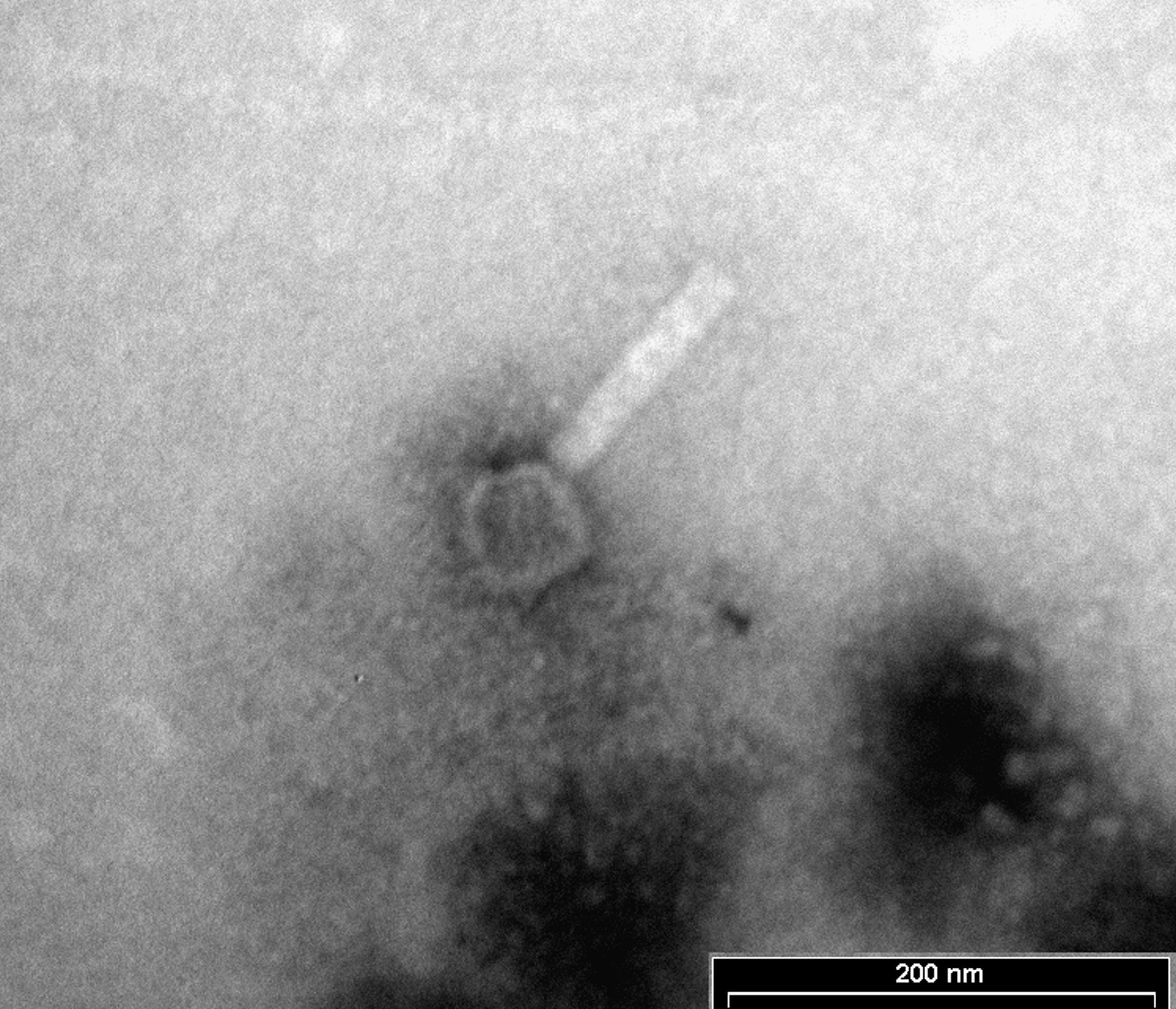

Supplement: Figure S1 — Phage particle of P. tunicata. Transmission electron micrograph of phage-like structures observed in the spent medium of Pseudoalteromonas tunicata. Bar = 200 nm (4.80 MB TIF) [file pone.0003252.s001.tif]
